# Desflurane Preconditioning Induces Oscillation of NF-κB in Human Umbilical Vein Endothelial Cells

**DOI:** 10.1371/journal.pone.0066576

**Published:** 2013-06-17

**Authors:** Juan Yi, Yijun Zheng, Changhong Miao, Jianguo Tang, Biao Zhu

**Affiliations:** 1 Department of Anesthesiology and Critical Care, Zhongshan Hospital, Fudan University, Shanghai, P.R. China; 2 Department of Anesthesiology, Shanghai Cancer Hospital, Fudan University, Shanghai, P.R. China; 3 Department of Emergency and Critical Care Unit, The Fifth People’s Hospital of Shanghai, Fudan University, Shanghai, P.R. China,; University of Cincinnati, United States of America

## Abstract

**Background:**

Nuclear factor kappa B (NF-κB) has been implicated in anesthetic preconditioning (APC) induced protection against anoxia and reoxygenation (A/R) injury. The authors hypothesized that desflurane preconditioning would induce NF-κB oscillation and prevent endothelial cells apoptosis.

**Methods:**

A human umbilical vein endothelial cells (HUVECs) A/R injury model was used. A 30 minute desflurane treatment was initiated before anoxia. NF-κB inhibitor BAY11-7082 was administered in some experiments before desflurane preconditioning. Cells apoptosis was analyzed by flow cytometry using annexin V–fluorescein isothiocyanate staining and cell viability was evaluated by modified tertrozalium salt (MTT) assay. The cellular superoxide dismutases (SOD) activitiy were tested by water-soluble tetrazolium salt (WST-1) assay. NF-κB p65 subunit nuclear translocation was detected by immunofluorescence staining. Expression of inhibitor of NF-κB-α (IκBα), NF-κB p65 and cellular inhibitor of apoptosis 1 (c-IAP1), B-cell leukemia/lymphoma 2 (Bcl-2), cysteine containing aspartate specific protease 3 (caspases-3) and *second* mitochondrial-derived activator of caspase (SMAC/DIABLO) were determined by western blot.

**Results:**

Desflurane preconditioning caused phosphorylation and nuclear translocation of NF-κB before anoxia, on the contrary, induced the synthesis of IκBα and inhibition of NF-κB after reoxygenation. Desflurane preconditioning up-regulated the expression of c-IAP1 and Bcl-2, blocked the cleavage of caspase-3 and reduced SMAC release, and decreased the cell death of HUVECs after A/R. The protective effect was abolished by BAY11-7082 administered before desflurane.

**Conclusions:**

The results demonstrated that desflurane activated NF-κB during the preconditioning period and inhibited excessive activation of NF-κB in reperfusion. And the oscillation of NF-κB induced by desflurane preconditioning finally up-regulated antiapoptotic proteins expression and protected endothelial cells against A/R.

## Introduction

Inhalation anesthetics have protective effects when administered before a period of ischemia and reperfusion (I/R), and this phenomenon is referred to as anesthetic preconditioning (APC) [1]. Researchers go in for studying the protective effect of APC in the two decades. Mechanism underlying APC is uncertain but believed to involve nuclear factor kappa B (NF-κB) pathway[2]–[5]. NF-κB proteins are classic rapid-acting transcription factors that regulate the expression of more than 200 target genes and play a pivotal role in several physiological process including immunity, inflammation, cell survival, differentiation and proliferation, and regulate cellular responses to stress, hypoxia, stretch and ischemia. The NF-κB family consists of five members: RelA (p65), c-Rel, RelB, p105/p50 (NF-κB1) and p100/p52 (NF-κB2). All members share an N-terminal Rel-homology domain (RHD), which is required for homo- and hetero-dimerization, nuclear translocation, association with inhibitory proteins and DNA binding. In unstimulated cells, NF-κB dimers are inactivated by binding to inhibitors of kappa B (IκBs), which sequester NF-κB in the cytoplasm by masking the nuclear localization sequence (NLS). In canonical activation pathway, various stimuli such as reactive oxygen species (ROS), interleukin-1 (IL-1), lipopolysaccharide (LPS) and tumor necrosis factor-α (TNF-α) can release NF-κB from I*κ*Bα (usually depends on I*κ*B kinase (IKK) regulated ubiquitination and degradation of I*κ*Bα), allowing it to transport to the nucleus. Upon nuclear translocation, activated NF-κB will bind to kappa B (κB) units in promoter or enhancer regions of the target genes to initiate transcription.

Nelson et al [6] showed TNF-α induced NF-κB (RelA) nuclear-cytoplasmic shuttling; this phenomenon was defined as oscillation of NF-κB translocation and confirmed by following multiple researches[7]–[9]. A delayed negative feedback loop drives oscillation of NF-κB between the nucleus and cytoplasm. NF-κB regulates transcription of self-inhibitor IκBα and zinc finger protein A20. IκBα transports NF-κB out of the nucleus by organizing the nuclear export sequence (NES), there by terminating NF-κB activation. A20 down regulates NF-κB by inhibiting IKK complex and blocking the degradation of IκBα.

Endothelial cells are the first barrier of vascular, and the integrity of vascular endothelium is the most important guarantee for maintenance of organ function. Dysfunction of vascular endothelium plays a pivotal role in the genesis and development of many diseases. Vascular endothelium presents as an important contributor to protect many organs against I/R injury [10]–[13]. The objective of our study was to determine whether desflurane preconditioning confers endothelial cells protection against A/R injury, and if so, whether NF-κB oscillation mediates this effect.

## Materials and Methods

### Cell Culture

The ethics committee of Zhongshan Hospital of Fudan University approved the protocol and written informed consents were obtained from the patients. Human umbilical vein endothelial cells (HUVECs) were isolated from human umbilical vein vascular wall using collagenase (Roche) treatment described by Bruno [14], and cultured in endothelial cell culture medium (ECM, ScienCell) with 5% fetal bovine serum (FBS), 1% endothelial cell growth supplement (ECGS), 100 U/ml penicillin and 100 µg/ml streptomycin sulfate in a humidified chamber containing 5% CO_2_ at 37°C Cells from passages 3–6 were used for experiments.

### Immunofluorescent Staining

**Figure 1:**
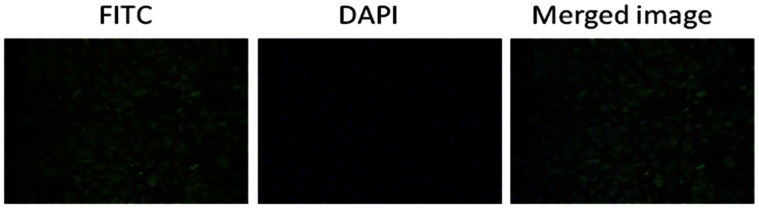
HUVECs immunofluorescence stained with anti-factor VIII related antibody. Column 1 is the image of factor VIII (green) in cytoplasm, column 2 is the image of nuclei (blue) and column 3 is the merged image. Magnification ×40.

Immunofluorescence staining was used to detect the endothelial cell marker factor VIII (Fig. 1) and NF-κB p65 subunit nuclear translocation. For immunofluorescent staining, cells grown on glass cover slips were fixed with 4% paraform, permeabilized with 0.1% Triton X-100, and blocked with 4% goat serum for 1 h. Cover slips were incubated overnight at 4°C in primary antibody solution (rabbit anti-factor VIII -related antigen, rabbit anti-NF-κB p65), then washed and incubated with secondary antibody (FITC-conjugated goat anti-rabbit IgG) for 1 h in 37°C and stained with 5 ng/ml DAPI to visualize the nucleus for 5 min, followed by further washes. Cover slips were mounted on microscope slides, and fluorescence was visualized with the microscope.

### Experimental Protocol

HUVECs were divided into 8 groups: control group (CON group), deflurane group (DES group), desflurane preconditioning+anoxia and reoxygenation group (DES+A/R group), anoxia and reoxygenation group (A/R group), BAY11-7082+ control group (BAY+CON), BAY11-7082+ desflurane group (BAY+DES group), BAY11-7082+ desflurane preconditioning+anoxia and reoxygenation group (BAY+DES+A/R group) and BAY11-7082+anoxia and reoxygenation group (BAY+A/R group).

**Figure 2:**
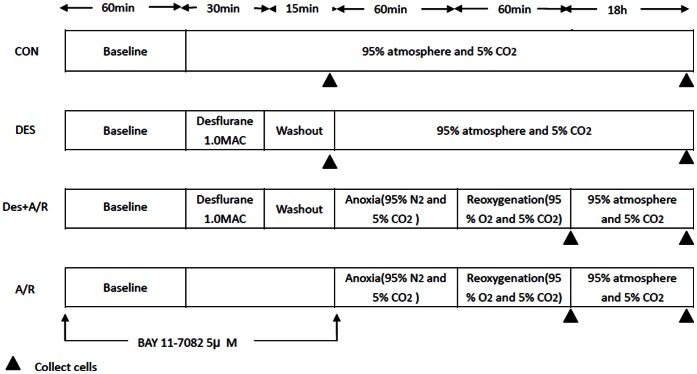
Experiment protocols. HUVECs submitted to the anoxia and reoxygenation (A/R) and pretreated with and without 1.0 minimum alveolar concentration (MAC) desflurane or 5 µM BAY11-7082 (BAY). For cell viability, apoptosis, SOD activity assays, the cell samples were collected at the end of experiments. For western blot analysis, cell samples were collected at the end of reoxygenation (samples were collected at 45 min after baseline in CON group and 15 min after desflurane exposure in DES group).

Cell monolayer exposed to A/R injury in an air tight chamber in a 37°C incubator. An infrared gas analyzer (Dräger) was used to continuously monitor the concentrations of carbon dioxide, oxygen, and desflurane at the gas outlet. Anoxia was performed by constantly ventilating 95%N_2_-5%CO_2_ gas mixture to the chamber to maintain the oxygen concentration lower than 2% for 1 hour, and then oxygenated by replacing with carbogen (95%O_2_-5%CO_2_) for 1 hour. In the DES+A/R group, cells were pretreated with 1.0 minimum alveolar concentration (MAC) desflurane for 30 min followed by a 15 min washout period before A/R injury. The concentration of desflurane was used to mimic the alveolar concentration in general anesthesia. BAY11-7082 is a kind of NF-κB inhibitor, which can inhibit IκBα degradation. Available researches and our preliminary experiment showed BAY 11-7082 in low-does can partially inhibit NF-κB activity without inducing significant cell apoptosis. In this research, 5 µM BAY11-7082 was added to the culture medium 1 hour before desflurane preconditioning (Fig. 2).

### Cell Viability Assay

The MTT assay was used because tetrazolium salts are cleaved to form a formazan dye only by metabolically active cells and are especially useful for assaying the quantification of viable cells. HUVECs were seeded in 96-well plates (3×10^4^ cells per well) and incubated overnight for complete cell adhesion. Then next day, A/R injury and desflurane preconditioning were performed as designed. At the end point of experiments, MTT (50 ul per well) was added to the medium and incubated at 37°C for a further 4 hours. Medium was removed from all wells and the insoluble formazan product was dissolved in 150 ul DMSO for 10 min at room temperature. Optical density (O.D.) of each culture well was measured using a spectrophotometer at 550 nm. The O.D. of control group cells represented 100% viability.

### Cell Apoptosis Assay

Cell apoptosis was detected by flow cytometry. The translocation of phosphatidylserine from the inner to the outer leaflet of the plasma membrane is an early event in apoptosis, and the binding of annexin V-fluorescein isothiocyanate (FITC) to phosphatidylserine in a Ca^2+^ dependent manner is used as a sensitive measure of apoptosis. Cells were double-stained with annexin V- FITC and propidium iodide (PI) according to the manufacturer’s instructions, and cell fluorescence was analyzed on a FACSan flow cytometer. Annexin V - FITC positive cells reflected the relative proportion of apoptotic cells.

### Cell Lysate Preparation

At the time points indicated in the experimental protocols, cells were scraped from the plates into ice-cold RIPA lysis buffer and total protein was collected in the supernatant.

Cytosolic protein without mitochondria was separated by differential centrifugation. Cells were harvested, washed with PBS, resuspended at in extraction buffer containing 250 mM sucrose, 1.5 mM MgCl_2_, 10 mM KCl, 25 mM Tris-HCl, 1 mM EDTA supplemented with additional protease inhibitors. Homogenized cells and confirmed 50% cells breakage has occurred. The homogenate was centrifuged at 600 g for 10 min. Supernant was collected and centrifuged at 12000 g for 20 min. Carefully aspirated supernant (cytosolic protein).

Protein concentration was measured with the bicinchoninic acid (BCA) method. Cell lysates were aliquoted and stored at −80°C until use.

### Measurement of Intracellular SOD Activity

The activity of SOD was determined using commercially available WST-1 assay kits. All procedures complied with the manufacturer’s instructions. The assay was based on its ability to inhibit the oxidation of hydroxylamine by O^2−^ produced from the xanthine–xanthineoxiase system. One unit of SOD activity was defined as the amount that reduced the absorbance at 450 nm by 50%. The results of SOD assay were expressed as units per milligram protein (U/mg protein).

### Western Blotting Analysis

For western blot analysis, denatured proteins (40 µg) were separated by 10% or 12% SDS-PAGE and transferred to polyvinylidene difluoride (PVDF, Millipore) membranes. Membranes were blocked with 5% no-fat milk in TBST for 2 h in room temperature and incubated with a rabbit monoclonal antibody recognizing c-IAP1, a rabbit monoclonal antibody recognizing Bcl-2, a rabbit monoclonal antibody recognizing IκB-α, a rabbit monoclonal antibody recognizing phosphor-NF-κB p65 (Ser 536), a mouse monoclonal antibody recognizing SMAC and a rabbit monoclonal antibody recognizing caspase-3 (1∶1,000 dilution, Cell Signaling Technology) at 4°C overnight. After washing in Tris-buffered saline Tween 20 (TBST), the blots were incubated with a secondary antibody (goat anti-rabbit IgG or goat anti-mouse IgG, 1∶1,000 dilution, Cell Signaling Technology) coupled to horseradish peroxidase (HRP) for 1 hour at room temperature. The blots were washed again in TBST buffer, and protein bonds were detected using enhanced chemiluminescent (ECL) reagent. Western blots of phosphor-NF-κB p65 were stripped and probed again with a rabbit monoclonal antibody recognizing NF-κB p65 (1∶1,000 dilution, Cell Signaling Technology). Tubulin and β-actin were used as an equivalent loading control.

The band densities were quantified using National Institutes of Health Image J software. Band densities for protein of interest were normalized to that of the band for tubulin or β-actin in the same sample.

### Statistical Analysis

GraphPad Prism 5 software was used to perform statistical analysis. All values were expressed as mean ± SD. Statistical comparisons were performed using one-way analysis of variance. Unpaired t test was used for comparisons between two groups. All *P* values were two-tailed, and *P*<0.05 was considered statistically significant.

## Results

### Desflurane Preconditioning Attenuated A/R Induced HUVECs Injury

**Figure 3:**
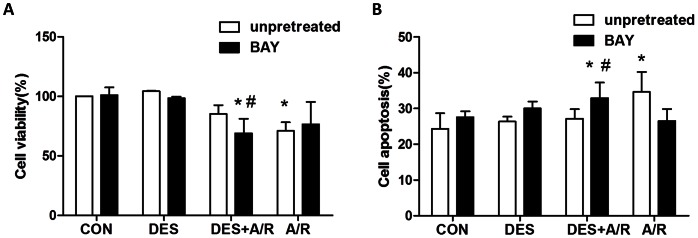
Effect of desflurane preconditioning on HUVECs submitted to A/R. Cells were submitted to A/R with or without pretreated with desflurane for 30 min or 5 µM BAY11-7082 (BAY). The viability was measured by MTT assay (A). The apoptosis rate was measured by FACSan flow cytometry (B). Data are presented as mean±SD. n = 6/group. *P<0.05 versus CON group,^ #^ *P*<0.05 versus DES+A/R group.

Figure 3A shows the viability of cells preconditioned with desflurane before submitted to A/R, as evidenced by MTT assay. A/R decreased the cell viability to 71.12±7.12%, while preconditioning with desflurane significantly diminished A/R induced cell death, restoring the cell viability of DES+A/R group to 85.32±7.28% (*P* = 0.0066).

To evaluate whether desflurane preconditioning was protecting cells against apoptosis after A/R, the cells were stained with annexin V-FITC and PI. A/R (Fig. 3B) induced significantly cell apoptosis (34.63±5.56%, *P* = 0.0051) compared with the control group, and this was partially reversed by desflurane preconditioning (27.13±2.67%, *P* = 0.0139). These results indicate that desflurane preconditioning decreased HUVECs apoptosis caused by A/R injury.

### Pretreated with BAY11-7082 Abolished Protection of Desflurane Preconditioning

In this research 5 µM BAY11-7082 (a kind of NF-κB inhibitor) was added to the cell culture medium 1 hour before desflurane preconditioning to test the effects of BAY11-7082 on desflurane preconditioning. We again evaluated the viability and apoptosis rate of cells pretreated with BAY11-7082. Figure 3A shows that, cell viability decreased to 69.07±12.26% in BAY+DES+A/R group, which was significantly lower compared to DES+A/R group (*P* = 0.0191). BAY11-7082 alone had no effect on cell viability. This phenomenon was further confirmed by flow cytometry, using annexin V-FITC and PI staining. Cell apoptosis rate (Fig. 3B) in BAY+DES+A/R group was 32.92±4.37%, which was significantly increased compared to DES+A/R group (*P* = 0.0199). These results suggested that pretreated with BAY11-7082 abolished the desflurane preconditioning protection against A/R injury.

### NF-κB p65 Activated by Desflurane in Preconditioning Period and Feedback Inhibits Activity of NF- κB after A/R

**Figure 4:**
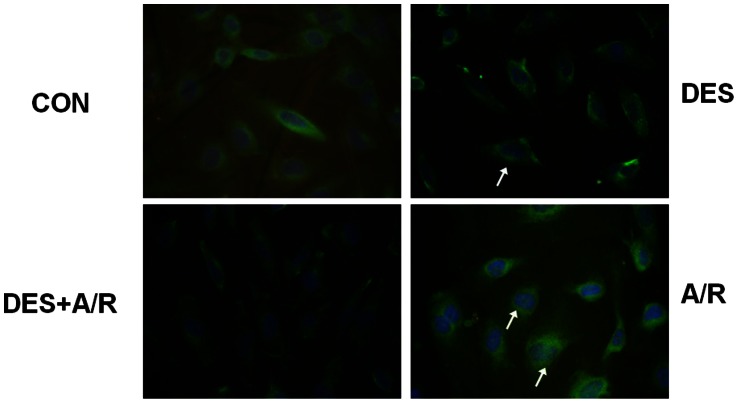
Effect of desflurane on NF-κB p65 nuclear translocation. Immunofluorescence assay showing NF-κB p65 subunit (green) translocated to nuclei (blue) after exposure to desflurane or A/R. Arrowheads signify some positive nuclei. Magnification ×200.

Given that NF-κB signaling pathway is involved in A/R induced cell apoptosis and oxidant injury [15], [16], we further investigated whether desflurane preconditioning affected the NF-κB pathway in endothelial cells submitted to A/R. We first stained NF-κB p65 with FITC and observed NF-κB p65 nuclear translocation in HUVECs in the absence or presence of desflurane. As shown in figure 4, desflurane or A/R injury alone rapidly induced NF-κB p65 nuclear translocation. Interesting, nuclear expression of NF-κB p65 in DES+A/R group cells was less than A/R group.

**Figure 5:**
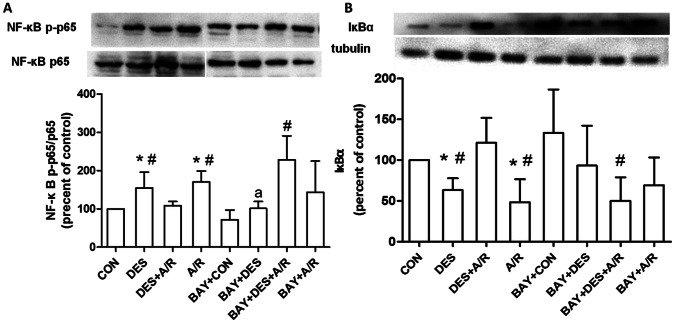
Western blotting and densitometry results. Western blot analysis showing concentration of phospho-NF-κB p-p65 and total NF-κB p65 (A), IκBα (B). Equal loading was confirmed by western blot with an anti-β-actin or anti-tubulin antibody. The IκBα, ratio of phospho-NF-κB p-p65 and total NF-κB p65 (p-p65/p65) protein concentrations were calculated by averaging the results obtained from six independent experiments. Data are presented as mean±SD. *P<0.05 versus CON group, ^#^ *P*<0.05 versus DES+A/R group, ^a^ *P*<0.05 versus DES group.

The degradation of IκBα induced NF-κB nuclear translocation, and the activity of NF-κB can be monitored by the phosphorylation of NF-κB p65 at serine 536 [17], [18]. We then examined the ratio of phospho-NF-κB p65 to total p65 (p-p65/p65) and the expression of IκBα in HUVECs before and after submitted to A/R with or without desflurane preconditioning. Figure 5 showed when compared to control, desflurane and A/R injury alone caused both NF-κB p65 phosphorylation (*P* = 0.0413 and 0.0051 respectively) and IκBα degradation (*P* = 0.0049 and 0.0149 respectively). Compared with A/R group, NF-κB p65 phosphorylation and IκBα degradation were significantly inhibited in DES+A/R group (*P* = 0.0018 and 0.0045 respectively). Desflurane preconditioning blocked the activation of NF-κB. These findings further confirmed that there was a negative feedback pathway of NF-κB involved desflurane preconditioning.

Before desflurane exposure, cells were pretreated with BAY11-7082 (BAY+DES group) demonstrated a blockage of NF-κB phosphrylation (*P* = 0.0288). While compared with DES+A/R group, BAY11-7082 increased both NF-κB p65 phosphrylation and IκBα degradation in BAY+DES+A/R group (P = 0.0028 and 0.0054 respectively). The result indicated that BAY11-7082 administered before desflurane abolished the attenuating effect of desflurane preconditioning on NF-κB overactivation when submitted to A/R.

### Desflurane Preconditioning Increases c-IAP1 and Bcl-2 Expression

**Figure 6:**
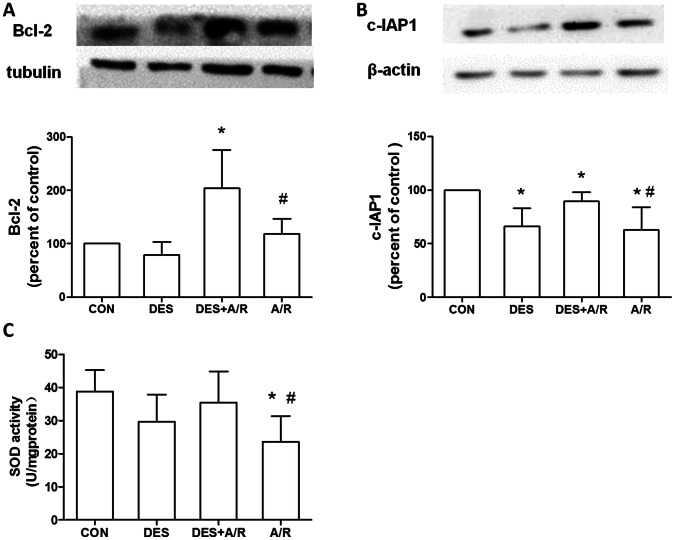
Effect of desflurane on expression of Bcl-2, c-IAP1 and SOD activity. Western blot analysis showing concentration of Bcl-2 (A), c-IAP1 (B). Equal loading was confirmed by western blot with an anti-β-actin or anti-tubulin antibody. The Bcl-2 and c-IAP1protein concentrations were calculated by averaging the results obtained from five independent experiments. The SOD activity was measured by WST-1 assay (C). Data are presented as mean±SD. *P<0.05 versus CON group, ^#^ *P*<0.05 versus DES+A/R group.

To gain insight into the effect of action of desflurane preconditioning induced NF-κB oscillation and cell protection, we thus examined whether desflurane preconditioning affects the expression of antiapoptotic protein c-IAP1 and Bcl-2, which are transcriptionally regulated by NF-κB. Compared A/R group (Fig. 6A), the expression of Bcl-2 in DES+A/R group significantly enhanced (*P* = 0.0382). Figure 6B showed compared to the control, c-IAP1 expression decreasing were observed in DES group, DES+A/R group and A/R group, but desflurane preconditioning before A/R injury (DES+A/R group) partially blocked the reduction of c-IAP1 caused by A/R injury (*P* = 0.0316). This result showed desflurane preconditioning increased c-IAP1 and Bcl-2 expression, which could be protective factors against anoxia and reoxygenation.

### Desflurane Preconditioning Increases SOD Activity

Superoxide dismutase (SOD) is an endogenous antioxidant, which can attenuate excessive ROS production and prevents post-ischemic injury [19], [20]. The effect of desflurane preconditioning on SOD was measured in this study (Fig. 6C). The intracellular SOD activity of control group was 38.86±6.40 U/mg proteins. Exposure to A/R injury significantly decreased SOD activity (23.55±7.84 U/mg protein, *P* = 0.0068), however desflurane preconditioning (DES+A/R group) reversed the injury, enhanced the SOD activity to 35.49±9.36 U/mg proteins (*P* = 0.0313). Preserved SOD activity confirms the protection of desflurane preconditioning.

### Desflurane Preconditioning Inhibits SMAC Release and Capases-3 Cleavage

**Figure 7:**
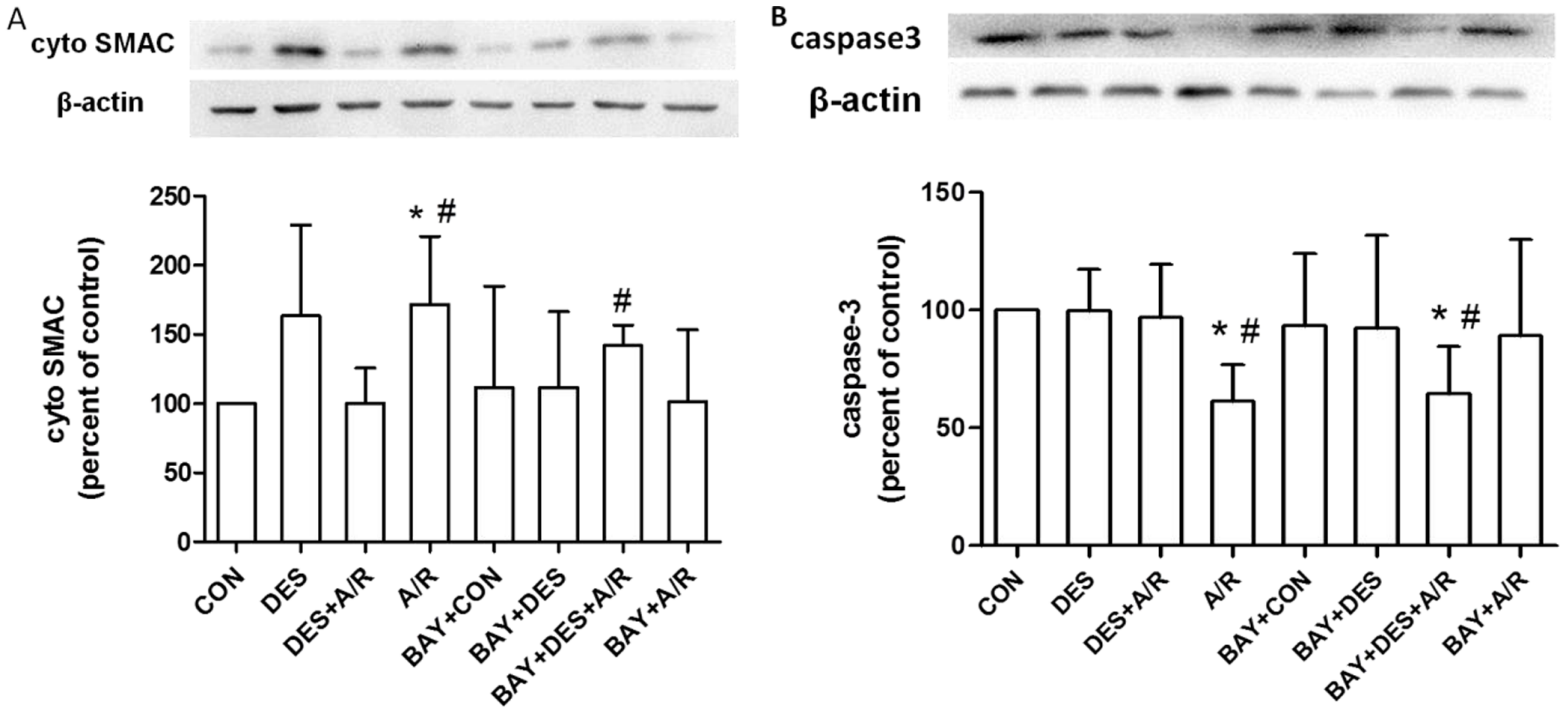
Eeffect of desflurane on SMAC release and capase-3 cleavage. Western blot analysis showing cytosolic SAMC (A) and total caspase-3 (B). Equal loading was confirmed by western blot with an anti-β-actin antibody. The cytosolic SMAC and total caspsase-3 concentrations were calculated by averaging the results obtained from five independent experiments. Data are presented as mean±SD. *P<0.05 versus CON group, ^#^ *P*<0.05 versus DES+A/R group.

Release of SMAC is known implicate in mitochondrial apoptotic pathway induced by most cell death-related stimuli. SMAC released into the cytosol triggers cell death by inhibiting the caspases inhibiting actions of the IAPs. By binding to IAPs, SMAC either displaces active caspases or prevents the binding of IAPs to active caspases, thus promoting cell death. Figure 7A showed A/R induced SMAC release (*P* = 0.0313 compared with CON group) which was reduced by desflurane preconditioning (*P* = 0.0205). When BAY11-7082 was added before desflurane, SMAC release was increased in BAY+DES+A/R group compared to DES+A/R group (*P* = 0.0133), which had no significant difference as compared to that of BAY +A/R (*P* = 0.1312).

Another protein implicated in apoptosis is caspase-3, a critical executioner of apoptosis, as it is either partially or totally responsible for the proteolytic cleavage of many key proteins such as the nuclear enzyme poly (ADPribose) polymerase (PARP) [21]. Activation of caspase-3 requires proteolytic processing of its inactive zymogen into activated p17 and p12 fragments [22]. We tested levels of full length of caspase-3 protein (35 kDa) in this experiment. HUVECs pretreated with desflurane showed significantly higher level of full-length caspase-3 after A/R than that without desflurane preconditioning (*P* = 0.0194, Fig. 7B), suggesting that desflurane preconditioning inhibited A/R induced caspase-3 cleavage and activation. When pretreated with BAY11-7082, caspase-3 level in BAY+DES+A/R group decreased compared to DES+A/R group (*P* = 0.0428) and had no significant difference compared to that of BAY+A/R group (*P* = 0.2650).

Therefore the above results indicate that mitochondrial apoptotic pathway is involved in desflurane induced NF-κB oscillation and cell protection. Moreover, inhibition of SMAC release and caspase-3 activation confirms desflurane-preconditioning protection against A/R injury in our cell model. Pretreated with BAY11-7082 abolished the deflurane preconditioning inhibition of SMAC release and capase-3 activation.

## Discussion

In the current experiments, we studied human umbilical vein endothelial cells which have similar bionomics with artery endothelial cells and testified that desflurane preconditioning induced protection against A/R injury by inhibiting cells apoptosis and preserving SOD activity, which is consistent with most published results [23]–[28]. In light of this, desflurane preconditioning can be considered as a potential therapy to use in condition where A/R caused endothelium dysfunction is implicated.

The major finding of this work is that desflurane induces NF-κB oscillation and finally increases antiapoptotic proteins transcriptionally regulated by NF-κB in HUVECs submitted to A/R injury. It is supported by the observations that desflurane preconditioning activated NF-κB before anoxia but inhibited overactivation of NF-κB after reoxygenation, and enhanced protein expression of c-IAP1 and Bcl-2. Previous studies, including ours, indicated that APC induced protection against A/R by inhibiting NF-κB activity [2], [23], [29]. Interesting, other researchers believed that APC induced protection though activation of NF-κB and synthesis of nitric oxide (NO) [30]. Lu and colleagues [31] investigated an isolated heart I/R model and validated that activation of NF-κB and expression of antiapoptotic proteins is a critical element of APC. Though the protective effect of APC is identified, it is still controversial whether inhalation anesthetics induced the protection by inhibiting or activating NF-κB.

NF-κB wildly presents in various cell types and function as the transcription factor of many genes such as cytokines, adhesion molecules, cyclins and antiapoptotic proteins. NF-κB plays a essential role in preventing cell apoptosis, also could induce cell death by increasing pro-inflammatory cytokines such as TNF, transforming growth factor-beta (TGF-β), enhancing sensitivity of cells to harmful stimulus and inhibiting expression of antiapoptotic proteins (x-IAP, Bcl-xL). Under normal circumstances, NF-*κ*B activation occurs rapidly and transiently. Dysregulated or constitutive NF-*κ*B activity, however, has been functionally linked to the development of a wide range of disorders such as carcinoma and I/R injury; activated NF-*κ*B, induced by pro-inflammatory cytokines (e.g. IL-1 and TNF-α) and endogenous ligands for Toll-like receptors (TLRs) that are generated in response to ischemia and the high level of ROS produced during reperfusion, enhancing even more pro-inflammatory cytokines and pro-apoptotic proteins that may cause tissue or organ injury. NF-κB activating stimuli lead to phosphorylation of Ser 32 and Ser 36 on IκBα and Ser 536 on NF-κB, then the phosphorylated IκBα is degraded by proteasome and liberates NF-κB dimer to translocate nucleus and target gene transcription. Newly synthesized free IκBα, one of the transcription targets of NF-κB, may bind to nuclear NF-κB, leading to export of the complex to the cytoplasm and terminating NF-κB activation. Our works demonstrate that desflurane induced NF-κB transporting to nucleus and synthesis of IκBα protein which feedback inhibited NF-κB activation in HUVECs submitted to A/R injury.

The functional consequences of NF-κB signaling depend on oscillation persistence and transcription of NF-κB target genes. Bcl-2 family and IAP (inhibitor of apoptosis) family are the critical protein families that targeted by NF-κB and involved in preventing A/R injury. Bcl-2 family plays a pivotal role in the regulation of mitochondrial outer membrane permeability (MOMP) [32]. Bcl-2 is a prototype antiapoptotic protein that localizes to the mitochondria and blocks the recruitment and activation Bax and Bad (pro-apoptotic protein of Bcl-2 family), thereby stabilizing mitochondrial membrane and inhibiting the release of apoptotic factors such as cytochrome c, *second* mitochondrial-derived activator of caspase (SMAC/DIABLO) and apoptosis-inducing factor (AIF) from the mitochondria. IAPs are a family of caspase inhibitors, providing a cytoprotective step downstream of death receptor or mitochondrial apoptosis. The IAP-IAP complex associates with executioner caspase-3 and -7, as well as initiator caspase-9 with high affinity, shutting off their cell killing ability. We find that desflurane preconditioning increased the expression of c-IAP1 and Bcl-2, inhibited cleavage of caspase-3 and leakage of SMAC in HUVECs submitted to A/R, accompanying attenuation of cell apoptosis. One possible explanation for the effect of desflurane on A/R injury may thus be its ability to induce NF-κB oscillation and finally up-regulate antiapoptotic protein c-IAP1 and Bcl-2 expression.

BAY11-7082, an inhibitor of NF-κB, has been demonstrated to reduce infarct size and preserve myocardial function in rat myocardial I/R injury model [33]. But current study on in vitro A/R model of HUVECs showed when administered before desflurane preconditioning, BAY11-7082 induced more cell death. We speculate that the possible reason that BAY11-7082 abolished desflurane protection is that it blocked NF-κB activation in preconditioning period; with blunted NF-κB activity, HUVECs synthesis insufficient IκBα when exposure to anoxia. And lacking of IκBα makes HUVECs can not resist overactivation of NF-κB caused by A/R injury. The result validated that previous activated NF-κB conduced to inhibiting overactivation of NF-κB and cell death after A/R injury.

**Figure 8:**
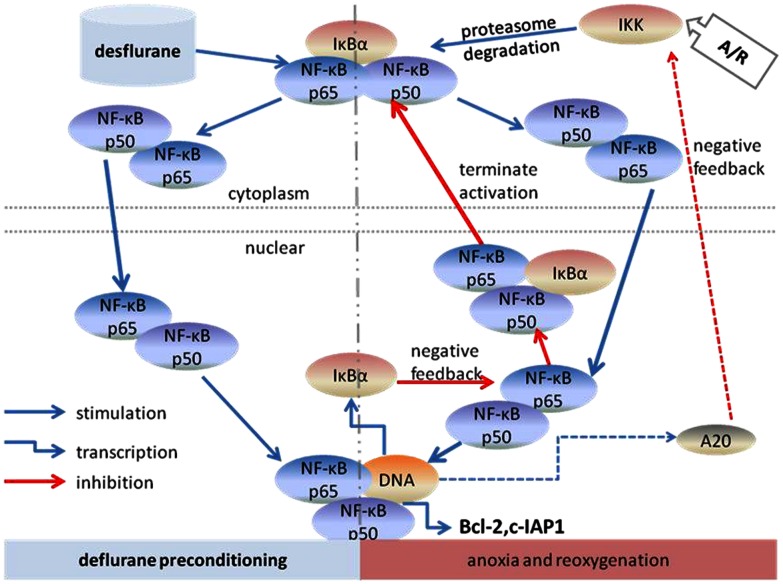
Desflurane preconditioning induced oscillation of NF-κB between nucleus and cytoplasm. Broken arrow refers to unconfirmed part of current study.

In conclusion, we have found that desflurane activated NF-κB during the preconditioning period, inhibited excessive activation of NF-κB in reperfusion, up-regulated Bcl-2 and c-IAP1 expression and inhibited SMAC release and caspase-3 cleavage after A/R injury. The data obtained in the present study suggest that the negative feedback induced oscillation of NF-κB translocation and up-regulation of antiapoptotic proteins plays a critical role in desflurane preconditioning induced protection in the vitro A/R model of HUVECs (Fig. 8). The oscillation of NF-κB may identify at least partially the molecular mechanism responsible for anesthetic preconditioning. Given that A/R induced NF-κB activation can lead to immune and inflammatory imbalance and cause cell apoptosis, the finding from the current studies will likely promote more studies aimed at determining the mechanism of APC induced protection and searching for prevent measures for A/R injury. Ultimately, these efforts will lead to better anesthesia care to patients.
